# Correction: A novel cytoskeletal action of xylosides

**DOI:** 10.1371/journal.pone.0305286

**Published:** 2024-06-21

**Authors:** Caitlin P. Mencio, Sharada M. Tilve, Masato Suzuki, Kyohei Higashi, Yasuhiro Katagiri, Herbert M. Geller

The fourth author’s name is spelled incorrectly. The correct name is: Kyohei Higashi. The correct citation is: Mencio CP, Tilve SM, Suzuki M, Higashi K, Katagiri Y, Geller HM (2022) A novel cytoskeletal action of xylosides. PLOS ONE 17(6): e0269972. https://doi.org/10.1371/journal.pone.0269972.
