# Supplementary material for: A novel cytoskeletal action of xylosides
Source: PLoS One. 2022 Jun 28;17(6):e0269972. doi: 10.1371/journal.pone.0269972 (PMC9239447; doi:10.1371/journal.pone.0269972)
Supplement: S1 Fig — A) Images of hippocampal neurons treated with either DMSO or LCX. LCX-treated neurons have large growth cones with extensive microtubule looping (arrow). B) Dose response curve for xyloside treatment. Percentage of neurons with looped microtubules at the end of the growth cones increased and peaked at 500 nM xyloside treatment. (PDF) [file pone.0269972.s001.pdf]

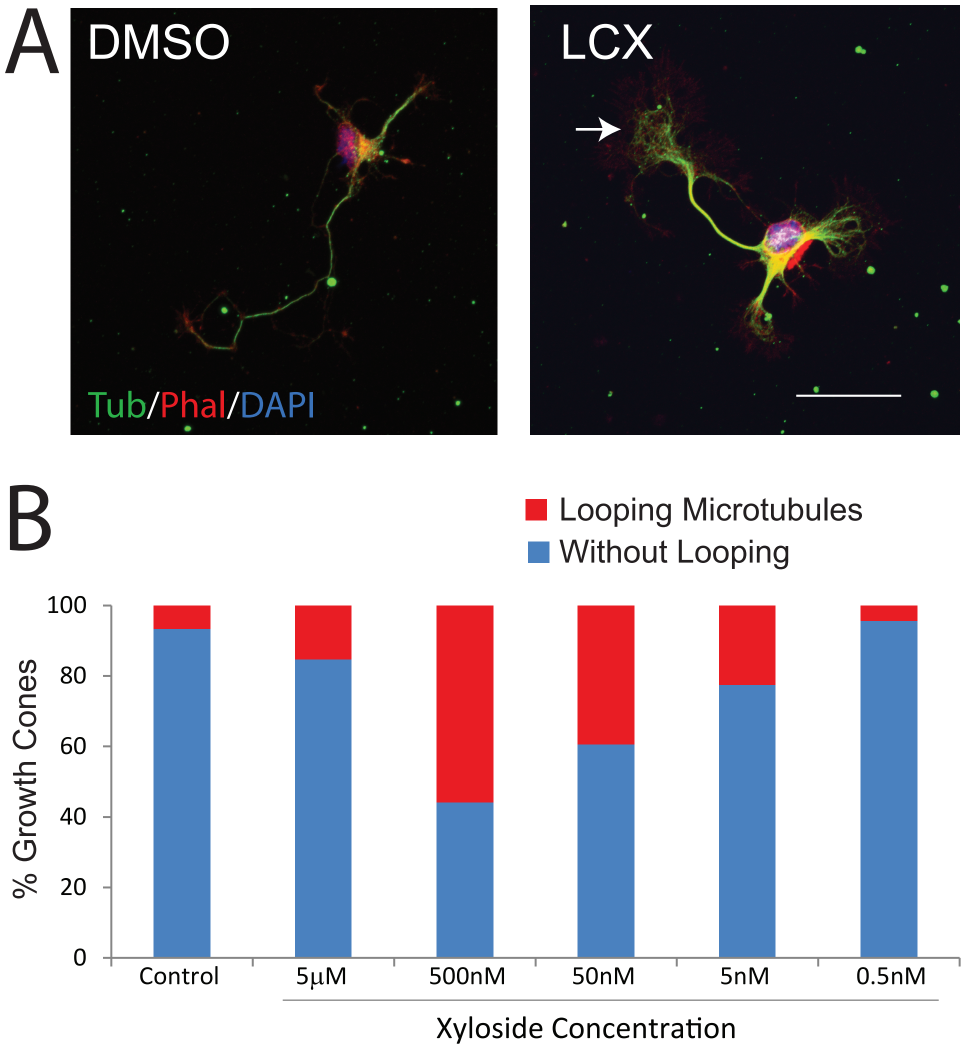

Supp. Figure 1. Microtubule looping in xyloside-treated growth cones. A) Images of hippocampal neurons treated with either DMSO or LCX. DMSO treated neurons have small actin-rich growth cones. LCX-treated neurons have large growth cones with extensive microtubule looping (arrow). B) Concentration-response curve for xyloside treatment. Percentage of neurons with looped microtubules at the end of the growth cones increased and peaked at 500 nM xyloside treatment.
