# Supplementary material for: A novel cytoskeletal action of xylosides
Source: PLoS One. 2022 Jun 28;17(6):e0269972. doi: 10.1371/journal.pone.0269972 (PMC9239447; doi:10.1371/journal.pone.0269972)
Supplement: S2 Fig — Images of hippocampal neurons treated with either DMSO, LCX or HCX 24 h after plating. Arrows point to splayed tubulin at the ends of growing neurites in LCX-treated cultures. Scale bar = 25 μm. (PDF) [file pone.0269972.s002.pdf]

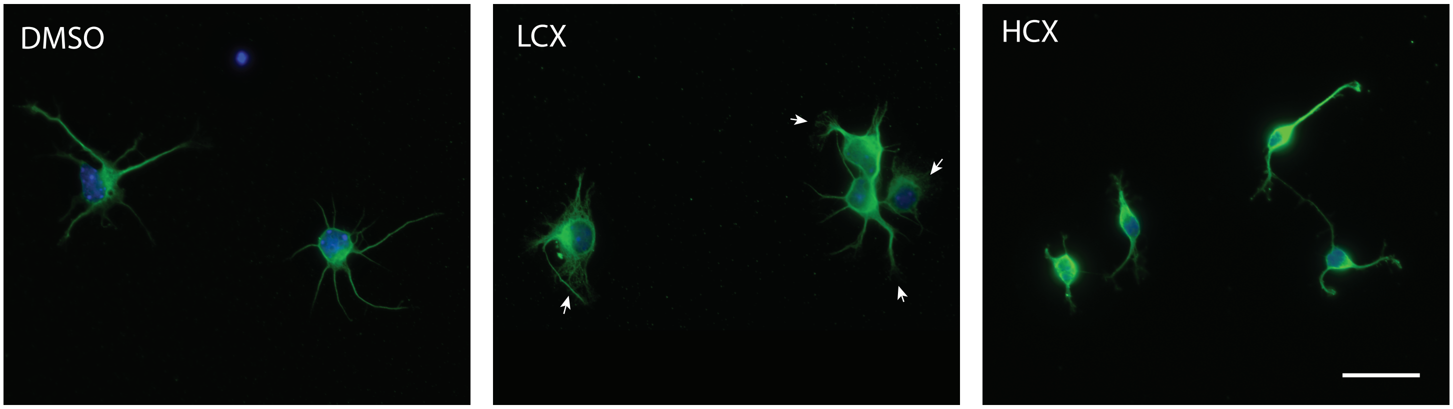

Supp. Figure 2. LCX treatment alters early neurite outgrowth. Images of hippocampal neurons treated with either DMSO, LCX or HCX 24 h after plating. Arrows point to splayed tubulin at the ends of growing neurites in LCX-treated cultures. Scale bar = 25 μm.
