# Supplementary material for: A novel cytoskeletal action of xylosides
Source: PLoS One. 2022 Jun 28;17(6):e0269972. doi: 10.1371/journal.pone.0269972 (PMC9239447; doi:10.1371/journal.pone.0269972)
Supplement: S3 Fig — Cells were transfected with F-tractin (red) and fixed and stained with DAPI (blue) 48 h later. (Left) DMSO-treated Neuro2a cells show typical morphology irregular shape and intense actin staining at the periphery. (Center) LCX-treated cell shows large lamellipodia (arrows) with centripetal actin organization. (Right) HCX-treated cells resemble DMSO-treated cells with irregular shape and peripheral actin staining. Scale = 25 μm. (PDF) [file pone.0269972.s003.pdf]

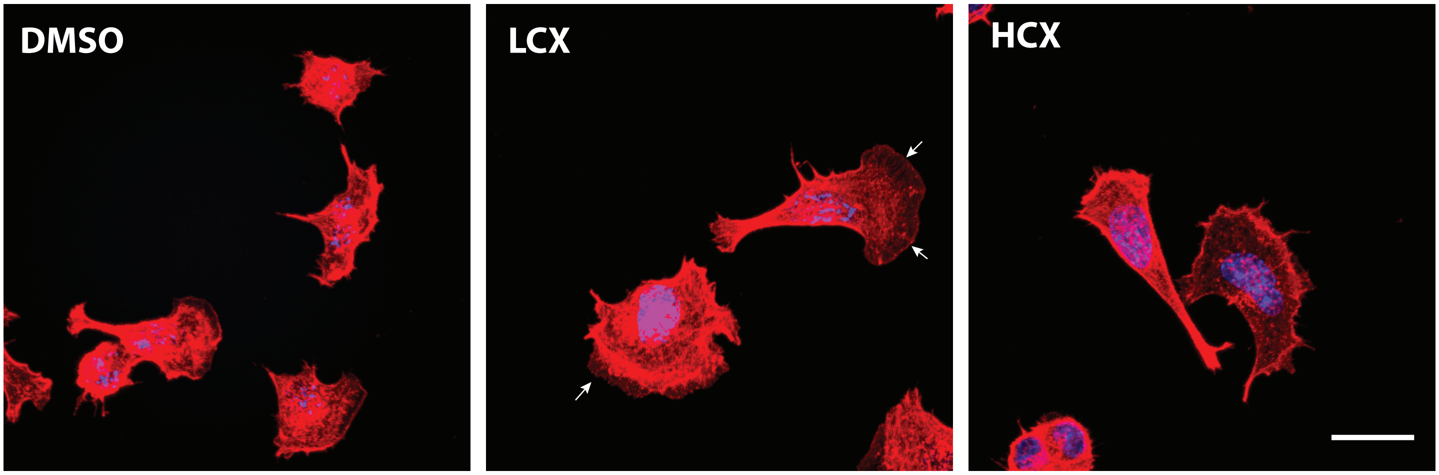


Supp. Figure 3. Altered Neuro2a morphology in cells treated with LCX. Cells were transfected with f-tractin (red) and fixed and stained with DAPI (blue) 48 h later. (Left) DMSO-treated Neuro2a cells show typical morphology irregular shape and intense actin staining at the periphery. (Center) LCX-treated cell shows large lamellipodia (arrows) with centripetal actin organization. (Right) HCX-treated cells resemble DMSO-treated cells with irregular shape and peripheral actin staining. Scale = 25 μm.
