# Supplementary material for: A novel cytoskeletal action of xylosides
Source: PLoS One. 2022 Jun 28;17(6):e0269972. doi: 10.1371/journal.pone.0269972 (PMC9239447; doi:10.1371/journal.pone.0269972)
Supplement: Attachment — Submitted filename: Re-review Mencio et al.pdf [file pone.0269972.s007.pdf]

The reviewer acknowledges that the revised ms has been profoundly reworked and greatly improved, although I am still not completely convinced by all arguments provided.

- I agree that the GAG chain synthesis and composition studies, and transfections, are easier to perform with N2A cells, but for the rest I personally do not see why hippocampal (or cortical? or maybe even adult DRG) neuron cultures are "too variable" as you say. Since your group is working on axon guidance, regeneration, etc., and you show here at the beginning of your ms how low dose xyloside treatment affects MT looping, growth cone stalling and neuronal morphology, I wonder how you will be able to "translate" results from your in vitro work with N2A cells lacking growth cones and even morphologically not really resembling neurons, for use in your other studies. Sure this is a personal "regret" of the reviewer, not relevant for publication in PLOSOne.

- You say that for Fig.1 you added images of several neurons at low power, but those are not found in the revised ms? Could be a Supplemental Figure.
- Supp Fig.2: Overall, there is now more similarity with Fig.3. However, the caption on the fig. does not correspond to the legend in the ms (line 584). At the same time, the caption on the fig. describes the facts better than the ms legend ("increased levels of interior ~~phalloidin~~ actin staining" vs. "increased levels of actin"; "actin" should at least read F-actin by the way). I think the legend to Supp Fig.2 should be more precise to not induce us into erroneous interpretation of Fig.3, which has obviously not changed (?).
- The discussion has been indeed improved. It does however not deal with the question where the effect on the actin cytoskeleton of LCX vs. HCX treatment may attack. Could there be an effect already during GAG synthesis in Golgi/cytosol and subsequent PG transport, or is there only an effect on (extracellular) signaling via secreted or membrane-bound PGs? (Let's say that is a question that would interest me personally, but you need not answer it).

- There are still some errors and typos, see below.

Line numbers refer to the Word (.docx) document "Final Revision" !
Abstract: the reformulated lines 29-31 (-32) are not very "elegant", and the end may be misleading ("higher concentrations had minor effects"). I'd propose something like:
To our surprise, we found that concentrations of xylosides in the nanomolar to micromolar range had major effects on cell morphology of hippocampal neurons as well as of Neuro2a cells, affecting both actin and tubulin cytoskeletal dynamics. Such effects/morphological changes were not observed with higher xyloside concentrations.

Line32: Xylosides ... produces...

Line33: ...large change in GAG chain synthesis rate

Finally, you did not include the effect on GAG composition in the Abstract, why? You have done so in the Introduction, where you state that your study may contribute to understanding "how a minor shift in GAG composition can affect biological processes..."

Cell culture: Stock solutions are now described, but it would not have been necessary to do it two times (lines 93, 107).
Line108: 10 m should probably read 10 min.

Growth cone analysis:
Lines164-167: this is a bit unclear for the reader. "The randomized files were then numbered sequentially and saved for reference. Duplicates of these files had all identifying information removed and then the numbered files were analyzed."
I guess that the duplicate files without information were analyzed (to make for 'double blind')? Here it sounds as if the reference files (the "numbered" ones) were analyzed. I'd prefer a simple: "Analysis was then performed on duplicates of these files from which all identifying information had been removed".

Lines162- : this does not really answer my question how "collapsed" GC were counted? (as seen in Fig.1: LCX shows a neat, large GC, DMSO an almost collapsed, and HCX no visible GC at all, making it impossible to count/evaluate microtubules).

Results:
Not very "elegant" beginning: "we sought to..., but we sought to..."; and the first sentence is not really true since you did not want to inhibit GAG synthesis here. Maybe you could start with something like "Previous studies on GAG chain synthesis had used...
Here, we wanted to establish a dose-response curve... to determine...".

Fig.1: You could have at least added F-actin staining of those "real" neurons since the rest of the paper is mostly about lamellae and F-actin on N2A cells. F-actin is shown in Supp Fig.1, but there an HCX image is missing.

Fig.2: As in the text you say that velocity and total distance were significantly different, this should be shown in the figure (that shows only velocity), or at least the significance values for total distance mentioned in the text.

Fig.3: the image selected in Supp Fig.2 is a bit closer in comparison now, but I'm still not convinced: what exactly do you designate lamellipodium here in Fig.3 (clearly identifiable in the Supp. Fig.2 for the LCX cell). We should see (if I get it right??) that in LCX treated cells there are well-formed lamellipodia (reminiscent of neural growth cone), but less and thinner actin bundles than in HCX cells.
Several typos in Supp Fig.2 legend on the figure itself (but not in the manuscript).

Line 273: (Figure 2C) should read Fig. 3C.

Discussion:
Lines 303-4: "Treatment with xyloside treatment..."
Line 408: I don't see how you can suggest a different action of LCX on hippocampal neurons and N2A cells based on MT looping in growth cones, since the latter don't form a growth cone (at least not in your study).
Line 442: ...caused changed...
Line 443: predominant effect of actin, or on actin?
Line 452: full stop missing.

Line 454: "...the phenomenology is dose dependent". Normally, phenomenology is a science (sort of) and cannot be dose dependent.
