# Supplementary material for: A novel cytoskeletal action of xylosides
Source: PLoS One. 2022 Jun 28;17(6):e0269972. doi: 10.1371/journal.pone.0269972 (PMC9239447; doi:10.1371/journal.pone.0269972)
Supplement: Attachment — Submitted filename: Review Mencio-xylosides.docx [file pone.0269972.s005.docx]

The ms by Mencio et al. describes how the cytoskeleton and the morphology of neural cells (here, hippocampal neurons and N2A cells) are affected by nanomolar doses of xylosides, compounds usually applied in the millimolar range to interfere with GAG chain synthesis. The work is principally interesting since it suggests that very subtle changes in GAG chain composition may affect the actin cytoskeleton and thus, cell morphology and migratory behavior. It is also another example of very low doses of a compound exhibiting different effects than high doses.

The reviewer regrets, however, that some findings of the authors seem contradictory, questionable or to the least, unclear for the reader (see below).

One main concern is that the discussion section, while exhaustively reporting on previous work on GAG chain modification by xylosides, tries hard but does not come up with a sort of theory, let alone an explanation for the low dose effect of xylosides described by the authors. Thus, for the reviewer, the conclusion of the study is already contained in the Introduction lines 69-71 ("this study serves as a first step...").

In the Discussion, any attempts to explain the observed effects are limited to "This is an area of research that needs further exploration", repeated several times. "... [low concentration] xyloside treatment may lean to subtle changes in GAG chains having more distinct biological effects" (lines 389-90), repeated just below (lines 406-408): "This could imply that utilizing lower concentrations that result in distinct, but subtle changes to GAG profiles may produce more unique and useful biological outcomes". Besides "more unique and useful" seeming rather "cryptic" terms in biology, these sentences don't explain, but simply repeat the observed findings. The fact that GAGs may interact for ex. with Sema5A (lines 382-386), which in turn may indeed affect the cytoskeleton, doesn't help either because Sema5A is not present here (as far as we know). Still other arguments are simply not new: (lines305-6): "These findings indicate that xyloside treatment can change GAG chain synthesis and composition, and alterations to HS appear to occur in a concentration dependent manner".

Maybe it could have helped if the authors had produced a model/schematic drawing of what they think happens on the molecular level when using low vs. high xyloside concentrations.

Another critical point may be seen in the fact that the authors used the same "LCX" concentration for primary hippocampus cells and for a "neural" cell line (N2A), with which the majority of the work was performed. Many studies have shown that different cell types react differently to a given concentration of xylosides, so it may have been interesting to redo a dose response curve with these cells.

Finally, one may ask whether the N2A cell line used in this study represents really neural cells (neurons), in view of the images shown that are very different from the hippocampal neurons shown in the beginning.

Major points:

Abstract line 32: "... did not alter GAG chain synthesis rates, nor..." the fact that GAG dissaccharide composition was altered is probably crucial and should be mentioned here.

Introduction, line 61: Other studies than the mentioned ref. 16 already used relatively low xyloside concentrations, for ex. Carrino & Caplan 1994 who used 1µM, which is not that far from 500nM (in comparison to mM).

Materials and Methods: for the reviewer, at least parts of this section are lacking some crucial details.

Controls for xyloside treatment are always termed "DMSO"; however, we do not know how much DMSO was introduced into the cultures, as we do not learn at what stock concentration xyloside was used. Other, similar studies always stated that they used the highest DMSO concentration for controls (corresponding to the highest xyloside conc. applied). So the reader may ask if DMSO addition already modifies something?

Cell culture line 83: cell density 8-10k/well (is this 12-well, 24-well, ...?).

24mM KCl was added to the culture of hippocampal neurons, why ? (usually this is done to depolarize the neurons, but here?).

Line 146- Neurite otgrowth and growth cone analysis: "both longest and total neurite measurements...": "total neurite" doesn't appear anywhere in the ms, and does it mean total length of all neurites ? How were neurite length, and the growth cone area exactly measured (ImageJ plugin?).

Cytoskeleton dynamics: EB3-GFP and Ftractin-mCherry transfections are not detailed (maybe a recent reference would suffice). Also, the reviewer asks whether, since transfections were performed after exposure to xyloside, are cells reacting the same to the transfections as without xyloside, what is the percentage of transfected cells, does transfection itself have other consequences on cell morphology ?

**Results:**

Figure 1: An overview image showing more than one neuron would be welcome; here, we just see one well spread growth cone (GC) formed by an LCX treated neuron, while the GC from control and DCX neurons appear rather collapsed. Also, the LCX neurite is much shorter than the other two, so I guess we are dealing with somewhat extreme (so called "typical") examples.

Supp Figure 1: to me, this is the "key" figure of the ms, as it shows the dose response curve for xyloside treatment, on which the rest of the ms, including the work on N2A, is based. The reviewer is puzzled when looking at the images: how many neurons do we see, particularly in the right LCX image? What looks like GC, contains also nuclei? Then, how were "looping MTs" quantified? In a "collapsed" GC like those seen on control neurons (left image) they would be hard to count. This is also not specified in M&M.

Supp Figure 2 is the basis for N2A cell line-based work. (Line 217) "... LCX treated N2A exhibit actin-rich lamellipodia, not observed in DMSO or HCX treated N2A". Besides the fact that in the LCX image the red color appears more saturated to me than in the other two images (see the completely flat yellow zones); this finding appears completely contradictory to Fig. 3 (see below).

Figure 2: Looking at the images, it seems strange to me that at t=0, the tracked cells do not show any extensions, which then develop at t=80 and t=160 min where cells really look like migratory cells (as if someone had given the "start signal"), although cells were kept under the same conditions already for 48h.

Figure 3: In the figure legend it says "phalloidin staining on fixed cells", in the text it says Ftractin mCherry transfection (normally used for live viewing?), what in fact do we see here ?

(lines 243-250) LCX treated N2A lamellipodia had significantly fewer actin bundles...; additionally, ...less robust actin bundles in LCX lammellipodia. How do the authors explain that in Supp Fig 2, DMSO or HCX treated N2A did not exhibit actin-rich lamellipodia, while here they state in some sort the opposite? Moreover, the cells in Fig. 3 do not resemble at all those shown in Supp Figure 2, let alone the (hippocampal) real neurons in Fig. 1. Here and elsewhere, figure legends (M&M) are not precise enough (different culture times between Supp Fig. 2 and Fig. 3, for example?).

To me, the HCX cell looks polarized, much more than the LCX cell; this is even more obvious in Figure 4A.

Figure 4: "These results [...] suggest LCX treatment alters cytoskeleton dynamics, which lead to changes in morphology and movement"; yes, but in Fig. 4 (EB3 comet movement) there is no difference between LCX and HCX treated N2A cells, only between DMSO and xyloside treatment. This is somehow in contrast to the assumption that low, but not high xyloside concentrations lead to major morphology changes (see e.g. Introduction line 64-65; Figs. 1, 2, Supp Fig2; discussion line 405-406: "no visible effect is observed in 1mM xyloside treated neurons or neuro2A cells").

Besides, as in Fig. 3, it looks to me that the cells presented differ in polarity, the HCX cell being the most polarized.

Figure 5: the legend should at least mention what means 0S, NS, 6S, 2SNS, especially for the broad audience of PlosOne who may not all be familiar with these terms.

The figure shows that CS GAG concentration in the medium is sort of xyloside dose dependent, whereas both low and high xyloside doses provoke the same change in CS disaccharide composition. HS GAG concentration in the medium is not altered by LCX, but the relative abundance of N-sulfated GAGs is. This seems interesting to me with regard to the role of N-sulfated HS reported in the literature (see e.g. Grobe et al 2005, Development 132: 3777), and this point could well be analyzed a little more in the Discussion section.

Another point: the difference between LCX effects on CS and HS GAG production may in fact be due to the DMSO controls already producing some HS (~0.1 ng/µL), but no CS.

The problem I see here, is that we do not learn whether the small effect of LCX on CS GAG concentration could have an effect on cell morphology, or whether the altered sulfation of HS GAGs does. These questions may partly be answered by, for example, rising (exogenous) CS concentration in the culture medium (i.e. not through xyloside treatment), or by selectively suppressing N-sulfation.

Figure 6C: there seem to be at least some genes whose expression is changed between DMSO and LCX ? The formulation (lines 314-15) "we found no genes whose change in expression was different between LCX and DMSO..." is not very clear.

Discussion:

When reading the discussion, two more points came to my mind: i) is the observed low dose effect on cell morphology specific for "neural" cells, or may it affect also the other cell types (muscle etc.) that had already been used in xyloside experiments? ii) It may have been interesting to include HS/CS staining of the cells after LCX/HCX treatment, as seen for ex. in the Nishimura et al. work (ref. 20).

Minor points:

The abbreviations for HS, CS (and KS) need be introduced (Introduction line 39).

Figure legends are often too short. Ex., Figure1: "altered cytoskeleton", the label is not indicated (tubulin?). Figure 2, "letters" should rather read "numbers". Figures 3 and 4, see before. Also in Fig.4, "distribution plots of speed, persistence and comets" ("lifetime" missing?), and in the histograms themselves is marked "200715_Speed" etc. which doesn't tell us anything. Figure 5, add abbreviations.

Supp Fig. 1, the bar is missing.

Some typo etc. errors, such as "DMOS" (line 104), "in in" (line 323), "on at" (line 344), "1C" (1µ?; line 346), "the change...were..." (line 356).
