# Supplementary material for: A novel cytoskeletal action of xylosides
Source: PLoS One. 2022 Jun 28;17(6):e0269972. doi: 10.1371/journal.pone.0269972 (PMC9239447; doi:10.1371/journal.pone.0269972)
Supplement: Attachment — Submitted filename: Response to Reviewers.docx [file pone.0269972.s006.docx]

Response to Reviewer :

We thank the reviewer for their thorough and incredibly valuable comments and suggestions on the manuscript. We appreciate that this is a novel observation and have tried to answer each of the reviewers’ comments with appropriate changes in either the text or the figures. We have organized this response such that each comment or suggestion has a reply in different font from the comment. We hope that this submission is suitable for publication.

Herbert M. Geller, for the authors.

The ms by Mencio et al. describes how the cytoskeleton and the morphology of neural cells (here, hippocampal neurons and N2A cells) are affected by nanomolar doses of xylosides, compounds usually applied in the millimolar range to interfere with GAG chain synthesis. The work is principally interesting since it suggests that very subtle changes in GAG chain composition may affect the actin cytoskeleton and thus, cell morphology and migratory behavior. It is also another example of very low doses of a compound exhibiting different effects than high doses.

The reviewer regrets, however, that some findings of the authors seem contradictory, questionable or to the least, unclear for the reader (see below).

One main concern is that the discussion section, while exhaustively reporting on previous work on GAG chain modification by xylosides, tries hard but does not come up with a sort of theory, let alone an explanation for the low dose effect of xylosides described by the authors. Thus, for the reviewer, the conclusion of the study is already contained in the Introduction lines 69-71 ("this study serves as a first step...").

In the Discussion, any attempts to explain the observed effects are limited to "This is an area of research that needs further exploration", repeated several times. "... [low concentration] xyloside treatment may lean to subtle changes in GAG chains having more distinct biological effects" (lines 389-90), repeated just below (lines 406-408): "This could imply that utilizing lower concentrations that result in distinct, but subtle changes to GAG profiles may produce more unique and useful biological outcomes". Besides "more unique and useful" seeming rather "cryptic" terms in biology, these sentences don't explain, but simply repeat the observed findings. The fact that GAGs may interact for ex. with Sema5A (lines 382-386), which in turn may indeed affect the cytoskeleton, doesn't help either because Sema5A is not present here (as far as we know). Still other arguments are simply not new: (lines305-6): "These findings indicate that xyloside treatment can change GAG chain synthesis and composition, and alterations to HS appear to occur in a concentration dependent manner".

Maybe it could have helped if the authors had produced a model/schematic drawing of what they think happens on the molecular level when using low vs. high xyloside concentrations.

We agree that the discussion was not as informative as we would all like. Unfortunately, we could not conduct the mechanistic studies that would be important to answer this question, and so any interprétations we have are speculative. We did not include a model/schematic, as we think that any figure would be based on these speculations, and a reader who simply looked at figures (as many do) could be misled. Instead, we have replaced the verbiage cited by the reviewer with a more focussed discussion about what our data do support and do not.

Another critical point may be seen in the fact that the authors used the same "LCX" concentration for primary hippocampus cells and for a "neural" cell line (N2A), with which the majority of the work was performed. Many studies have shown that different cell types react differently to a given concentration of xylosides, so it may have been interesting to redo a dose response curve with these cells.

While we began these studies with hippocampal neurons, we realized that certain data on biochemistry would be difficult to determine using primary cultures which can be highly variable. For consisency, we therefore evaluated the two concentrations of xylosides that were used in hippocampal neurons on Neuro2a cells and found that they had differential concentration-dependent effects at the same concentrations used in neurons, and therefore continued with these concentrations.

Finally, one may ask whether the N2A cell line used in this study represents really neural cells (neurons), in view of the images shown that are very different from the hippocampal neurons shown in the beginning.

Neuro2a cells are mouse neuroblastoma cells and have been used in many different settings to examine signal transduction and biochemistry. Because they are a tumor cell line, they may exhibit various morphologies depending upon treatment, as we show here. There are many published papers that present data from both hippocampal neurons and Neuro2A cells, but we do not presume that all measurements will be the same in both.

Major points:

Abstract line 32: "... did not alter GAG chain synthesis rates, nor..." the fact that GAG dissaccharide composition was altered is probably crucial and should be mentioned here.

We agree and have modified the manuscript both in the introduction and discussion to emphasize these results.

Introduction, line 61: Other studies than the mentioned ref. 16 already used relatively low xyloside concentrations, for ex. Carrino & Caplan 1994 who used 1µM, which is not that far from 500nM (in comparison to mM).

We did cite this paper and its results in the discussion, but we have now included it in the introduction as well.

Materials and Methods: for the reviewer, at least parts of this section are lacking some crucial details.

Controls for xyloside treatment are always termed "DMSO"; however, we do not know how much DMSO was introduced into the cultures, as we do not learn at what stock concentration xyloside was used. Other, similar studies always stated that they used the highest DMSO concentration for controls (corresponding to the highest xyloside conc. applied). So the reader may ask if DMSO addition already modifies something?

We have edited the methods to show the stock concentrations and the dilution factor for DMSO and xylosides used in the paper. Additionally, we had compared DMSO treatment to untreated cells and saw no noticable difference in morphology. No previous studies have shown any changes in the cell at the percentage of DMSO we utilize.

Cell culture line 83: cell density 8-10k/well (is this 12-well, 24-well, ...?).

We have clarified which cell culture dishes were used as well as cell densities for each type of dish.

24mM KCl was added to the culture of hippocampal neurons, why ? (usually this is done to depolarize the neurons, but here?).

This concentration of KCl added to neurobasal medium has been found to promote neuronal health and survival of cultured neurons, and we do this in our lab protocols (Pearson CS, Mencio CP, Barber AC, Martin KR, Geller HM. Identification of a critical sulfation in chondroitin that inhibits axonal regeneration. Elife. 2018 May 15;7:e37139. doi: 10.7554/eLife.37139. PMID: 29762123; PMCID: PMC5976435.) A literature search shows that there are many other publications using high KCl to promote neuronal culture survival.

Line 146- Neurite outgrowth and growth cone analysis: "both longest and total neurite measurements...": "total neurite" doesn't appear anywhere in the ms, and does it mean total length of all neurites ? How were neurite length, and the growth cone area exactly measured (ImageJ plugin?).

We have added more detail into the materials and methods section on the analysis and data collection for neurites and growth cones.

Cytoskeleton dynamics: EB3-GFP and Ftractin-mCherry transfections are not detailed (maybe a recent reference would suffice). Also, the reviewer asks whether, since transfections were performed after exposure to xyloside, are cells reacting the same to the transfections as without xyloside, what is the percentage of transfected cells, does transfection itself have other consequences on cell morphology ?

The tranfection order was determined based on optimization of both cell survival and transfection efficiency. We found routinely about 40% of cells were transfected. We have used this transfection protocol to express GFP (without xylosides) in other experiments with Neuro2A cells (Agbaegbu Iweka C, Hussein RK, Yu P, Katagiri Y, Geller HM. The lipid phosphatase-like protein PLPPR1 associates with RhoGDI1 to modulate RhoA activation in response to axon growth inhibitory molecules. J Neurochem. 2021 May;157(3):494-507. doi: 10.1111/jnc.15271. Epub 2021 Jan 3. PMID: 33320336; PMCID: PMC8106640) (now cited in the manuscript) and have not observed changes in morphology.

Results:

Figure 1: An overview image showing more than one neuron would be welcome; here, we just see one well spread growth cone (GC) formed by an LCX treated neuron, while the GC from control and DCX neurons appear rather collapsed. Also, the LCX neurite is much shorter than the other two, so I guess we are dealing with somewhat extreme (so called "typical") examples.

We have added images of several HC neurons at low power. Unfortunately, it is difficult to display the large growth cônes and looping at that magnification, so we have retained the higher res images.

Supp Figure 1: to me, this is the "key" figure of the ms, as it shows the dose response curve for xyloside treatment, on which the rest of the ms, including the work on N2A, is based. The reviewer is puzzled when looking at the images: how many neurons do we see, particularly in the right LCX image? What looks like GC, contains also nuclei?

Indeed, there were several neurons in each image as indicated by the DAPI staining, making it difficult to interpret. We have therefore replaced image in the figure with one of a single neuron with the representative phenotype. We hope that these images are more easily interpreted.

Then, how were "looping MTs" quantified? In a "collapsed" GC like those seen on control neurons (left image) they would be hard to count. This is also not specified in M&M.

We have edited our materials and methods to provide clarity on how we determined the presence of looped MTs.

Supp Figure 2 is the basis for N2A cell line-based work. (Line 217) "... LCX treated N2A exhibit actin-rich lamellipodia, not observed in DMSO or HCX treated N2A". Besides the fact that in the LCX image the red color appears more saturated to me than in the other two images (see the completely flat yellow zones); this finding appears completely contradictory to Fig. 3 (see below).

We appreciate this comment. N2A cells have various morphologies and our goal was to select representative images from cells grown at the same time, and so the cells in Supp. Fig. 2 were somewhat different from those in Fig. 3. We have now selected images of f-tractin transfected cells for Supp. Fig. 2 which are contemporaneous with those in Fig. 3. These images more clearly display the morphologies quantified in N2A cells.

Figure 2: Looking at the images, it seems strange to me that at t=0, the tracked cells do not show any extensions, which then develop at t=80 and t=160 min where cells really look like migratory cells (as if someone had given the "start signal"), although cells were kept under the same conditions already for 48h.

These images are included as examples of how we can track movement. Not all the cells are migrating all the time. However, we have replaced the former images with images showing the various morphologies.

Figure 3: In the figure legend it says "phalloidin staining on fixed cells", in the text it says Ftractin mCherry transfection (normally used for live viewing?), what in fact do we see here ?

We thank the referee for pointing this out. We have corrected the text to correspond with the figure.

(lines 243-250) LCX treated N2A lamellipodia had significantly fewer actin bundles...; additionally, ...less robust actin bundles in LCX lammellipodia. How do the authors explain that in Supp Fig 2, DMSO or HCX treated N2A did not exhibit actin-rich lamellipodia, while here they state in some sort the opposite? Moreover, the cells in Fig. 3 do not resemble at all those shown in Supp Figure 2, let alone the (hippocampal) real neurons in Fig. 1. Here and elsewhere, figure legends (M&M) are not precise enough (different culture times between Supp Fig. 2 and Fig. 3, for example?).

We hope that we have answered this comment with the images in the new Supp. Fig. 2 which clearly shows the actin bundles in the LCX-treated cells. We also provide more details on the culture and transfection time in the figure legends.

To me, the HCX cell looks polarized, much more than the LCX cell; this is even more obvious in Figure 4A.

We did not observe consistent changes in polarity. We have selected new representative images that show cells in a more similar state of polarization across all three experimental conditions.

Figure 4: "These results [...] suggest LCX treatment alters cytoskeleton dynamics, which lead to changes in morphology and movement"; yes, but in Fig. 4 (EB3 comet movement) there is no difference between LCX and HCX treated N2A cells, only between DMSO and xyloside treatment. This is somehow in contrast to the assumption that low, but not high xyloside concentrations lead to major morphology changes (see e.g. Introduction line 64-65; Figs. 1, 2, Supp Fig2; discussion line 405-406: "no visible effect is observed in 1mM xyloside treated neurons or neuro2A cells").

Besides, as in Fig. 3, it looks to me that the cells presented differ in polarity, the HCX cell being the most polarized.

Thank you for pointing out this issue. We have now modified the text in both the results and discussion to point out the difference in the response of the actin and tubulin cytosekeletons to xylosides, i.e., that both concentrations alter MT dynamics, but only LCX alters actin.

Figure 5: the legend should at least mention what means 0S, NS, 6S, 2SNS, especially for the broad audience of PlosOne who may not all be familiar with these terms.

The figure shows that CS GAG concentration in the medium is sort of xyloside dose dependent, whereas both low and high xyloside doses provoke the same change in CS disaccharide composition. HS GAG concentration in the medium is not altered by LCX, but the relative abundance of N-sulfated GAGs is. This seems interesting to me with regard to the role of N-sulfated HS reported in the literature (see e.g. Grobe et al 2005, Development 132: 3777), and this point could well be analyzed a little more in the Discussion section.

We agree that the these findings were not adequately discussed. We have altered the discussion of the GAG sulfation to also indicate biological implications We have also included the definitions in the figure legend as requested.

Another point: the difference between LCX effects on CS and HS GAG production may in fact be due to the DMSO controls already producing some HS (~0.1 ng/µL), but no CS.

The problem I see here, is that we do not learn whether the small effect of LCX on CS GAG concentration could have an effect on cell morphology, or whether the altered sulfation of HS GAGs does. These questions may partly be answered by, for example, rising (exogenous) CS concentration in the culture medium (i.e. not through xyloside treatment), or by selectively suppressing N-sulfation.

We agree that these are possibilities. Unfortunately, we are unable to conduct these experiments within a reasonable time frame, as the NIH has been quite strict about lab occupancy. We did think about adding the conditioned medium from HCX cells to the LCX condition, but this would still be contaminated by xylosides. In response to this and the suggestion above, we have included these possibilities in the discussion for potential future experiments.

Figure 6C: there seem to be at least some genes whose expression is changed between DMSO and LCX ? The formulation (lines 314-15) "we found no genes whose change in expression was different between LCX and DMSO..." is not very clear.

According to our analysis using the Partek Flow pipeline, there were no genes whose expression was significantly changed (FDR < 0.05) between the LCX and DMSO conditions. Fig. 6C included all genes whose expression was significantly different between either LCX and HCX or DMSO and HCX, but we put in the relative change in expression for all conditions. Thus, the top one, Gstp3, was only significantly changed between HCX and LCX.

Discussion:

When reading the discussion, two more points came to my mind: i) is the observed low dose effect on cell morphology specific for "neural" cells, or may it affect also the other cell types (muscle etc.) that had already been used in xyloside experiments? ii) It may have been interesting to include HS/CS staining of the cells after LCX/HCX treatment, as seen for ex. in the Nishimura et al. work (ref. 20).

It is clearly possible that other cell types may respond similarly. As noted in the manuscript, few papers used comparable concentrations of xylosides in their experiments, so there is no point of reference. As to the staining with HS/CS, be believe that the biochemical experiments are more informative, as they looked at both production and composition which is not possible with immunocytochemistry.

Minor points:

The abbreviations for HS, CS (and KS) need be introduced (Introduction line 39).

We have introduced these abbreviations

Figure legends are often too short. Ex., Figure1: "altered cytoskeleton", the label is not indicated (tubulin?).

Thanks, we have now indicated that this is tubulin staining, and added additional text to the other legends as well.

Figure 2, "letters" should rather read "numbers".

Thanks again. Corrected.

Figures 3 and 4, see before. Also in Fig.4, "distribution plots of speed, persistence and comets" ("lifetime" missing?), and in the histograms themselves is marked "200715_Speed" etc. which doesn't tell us anything.

We thank the reviewer for catching these errors. They have been corrected.

Figure 5, add abbreviations.

Done

Supp Fig. 1, the bar is missing. FIxed

Some typo etc. errors, such as "DMOS" (line 104), "in in" (line 323), "on at" (line 344), "1C" (1µ?; line 346), "the change...were..." (line 356).

Thank you. We hope this version is free of typos.
