# Supplementary material for: A novel cytoskeletal action of xylosides
Source: PLoS One. 2022 Jun 28;17(6):e0269972. doi: 10.1371/journal.pone.0269972 (PMC9239447; doi:10.1371/journal.pone.0269972)
Supplement: Attachment — Submitted filename: Response to reviewers.docx [file pone.0269972.s008.docx]

We really appreciate the time the reviewer has taken with this manuscript. We have made additional changes in response to the comments and questions, and have attempted to answer each of the points made by the referee. Because we consider these comments to have greatly improved the manuscript, we have added an acknowledgment of the referee. We hope that these changes have made the manuscript acceptable for publication in PLOS One.

The reviewer acknowledges that the revised ms has been profoundly reworked and

greatly improved, although I am still not completely convinced by all arguments

provided.

- I agree that the GAG chain synthesis and composition studies, and transfections, are

easier to perform with N2A cells, but for the rest I personally do not see why

hippocampal (or cortical? or maybe even adult DRG) neuron cultures are "too variable"

as you say. Since your group is working on axon guidance, regeneration, etc., and you

show here at the beginning of your ms how low dose xyloside treatment affects MT

looping, growth cone stalling and neuronal morphology, I wonder how you will be able

to "translate" results from your in vitro work with N2A cells lacking growth cones and

even morphologically not really resembling neurons, for use in your other studies. Sure

this is a personal "regret" of the reviewer, not relevant for publication in PLOSOne.

I would not argue that the questions you raise are important, just that we don’t have the resources to address them at this time. While the cells are quite different, the result that low concentrations of xylosides have specific actions on the cytoskeleton is likely to be a general effect on many different cell types (though we have not looked a many).

- You say that for Fig.1 you added images of several neurons at low power, but those are

not found in the revised ms? Could be a Supplemental Figure.

Unfortunately, low power images of 72-h treated neurons do not have adequate resolution for fine tubulin or actin filaments, as we plated at low density to avoid neurite overgrowth. However, we have added a supplementary figure showing that differences in neurite morphology could be observed as early as 24 h after plating. Because these cells had much shorter neurites, we are able to present images with more than one cell at a time at a lower resolution than in the other figures. I am also including a low power reviewers’ figure which does not have the adequate resolution to demonstrate the microtubule morphology, but does show general morphology. We hope that these additional images will help interpret the phenomenon.

- Supp Fig.2: Overall, there is now more similarity with Fig.3. However, the caption on

the fig. does not correspond to the legend in the ms (line 584).

At the same time, the caption on the fig. describes the facts better than the ms legend ("increased levels of interior phalloidin actin staining" vs. "increased levels of actin"; "actin" should at least read F-actin by the way). I think the legend to Supp Fig.2 should be more precise to not induce us into erroneous interpretation of Fig.3, which has obviously not changed (?).

The original images that were used to illustrate the technique in Fig. 3 were not representative of the morphology under LCX treatment. We have now replaced the Fig. 3 images with ones that are more representative. In addition, the supplemental figure is now Supp. Fig. 3 and we have corrected the figure legend. We think these should adequately illustrate the phenomenon.

- The discussion has been indeed improved. It does however not deal with the question

where the effect on the actin cytoskeleton of LCX vs. HCX treatment may attack. Could

there be an effect already during GAG synthesis in Golgi/cytosol and subsequent PG

transport, or is there only an effect on (extracellular) signaling via secreted or

membrane-bound PGs? (Let's say that is a question that would interest me personally,

but you need not answer it).

This is also a great question. I actually lean towards the idea that the xylosides might be acting on the cytoskeleton totally independentely of GAGs, since the effects of LCX are modest on GAG secretion (though they do change GAG composition). However, proving this is beyond the scope of the current manuscript, and I did not want to put idle speculation in the text.

- There are still some errors and typos, see below.

*Line numbers refer to the Word (.docx) document "Final Revision" !*

Abstract: the reformulated lines 29-31 (-32) are not very "elegant", and the end may be

misleading ("higher concentrations had minor effects"). I'd propose something like:

To our surprise, we found that concentrations of xylosides in the nanomolar to micromolar

range had major effects on cell morphology of hippocampal neurons as well as of Neuro2a

cells, affecting both actin and tubulin cytoskeletal dynamics. Such effects/morphological

changes were not observed with higher xyloside concentrations.

Thanks for taking so much time for this. We have reworded using your suggestions.

Line32: Xylosides ... produces...

Line33: ...large change in GAG chain synthesis rate

Finally, you did not include the effect on GAG composition in the Abstract, why? You

have done so in the Introduction, where you state that your study may contribute to

understanding "how a minor shift in GAG composition can affect biological processes..."

Cell culture: Stock solutions are now described, but it would not have been necessary to

do it two times (lines 93, 107).

We now include GAG composition I the abstract.

Line108: 10 m should probably read 10 min.

Growth cone analysis:

Lines164-167: this is a bit unclear for the reader. "The randomized files were then

numbered sequentially and saved for reference. Duplicates of these files had all identifying

information removed and then the numbered files were analyzed."

I guess that the duplicate files without information were analyzed (to make for 'double

blind')? Here it sounds as if the reference files (the "numbered" ones) were analyzed. I'd

prefer a simple: "Analysis was then performed on duplicates of these files from which all

identifying information had been removed".

We again thank the reviewer for suggesting better wording.

Lines162- : this does not really answer my question how "collapsed" GC were counted?

(as seen in Fig.1: LCX shows a neat, large GC, DMSO an almost collapsed, and HCX no

visible GC at all, making it impossible to count/evaluate microtubules).

We divided neurons into categories based on whether they contained looped microtubules or not in any of their growth cones, and did not focus on any other morphological features, including collapse. Low power images show that most growth cones had some actin staining suggesting that they are not collapsed, but these images do not have the resolution for further evaluation. We hope that the current presentation focusing on microtubules in growth cones is sufficient to demonstrate the phenomenon.

Results:

Not very "elegant" beginning: "we sought to..., but we sought to..."; and the first sentence

is not really true since you did not want to inhibit GAG synthesis here. Maybe you could

start with something like "Previous studies on GAG chain synthesis had used...

Here, we wanted to establish a dose-response curve... to determine...".

I’ve tried to rewrite this sentence, but it seems that being accurate is not entirely compatible with being elegant. So I changed it to “xyloside treatment”, rather than GAG chain inhibition.

Fig.1: You could have at least added F-actin staining of those "real" neurons since the

rest of the paper is mostly about lamellae and F-actin on N2A cells. F-actin is shown in

Supp Fig.1, but there an HCX image is missing.

Unfortunately, we concentrated on MT looping when we took these images, and so we did not have suitable images that contain F-actin for HCX at the equivalent power. I am including a reviewer’s figure that does have f-actin staining of hippocampal neurons, but these images were taken at 10X, such that microtubule looping is poorly imaged. But it does show f-actin p-domains in most neurites. We would be happy to include this figure if the reviewer thought it will help.

Fig.2: As in the text you say that velocity and total distance were significantly different,

this should be shown in the figure (that shows only velocity), or at least the significance

values for total distance mentioned in the text.

We have now included the total distance in the results, though the result is not really independent since the persistence was not significantly different.

Fig.3: the image selected in Supp Fig.2 is a bit closer in comparison now, but I'm still not

convinced: what exactly do you designate lamellipodium here in Fig.3 (clearly

identifiable in the Supp. Fig.2 for the LCX cell).

We should see (if I get it right??) that in LCX treated cells there are well-formed lamellipodia (reminiscent of neural growth cone), but less and thinner actin bundles than in HCX cells.

We agree that the images were not optimal As noted above, we have replaced the images in Fig. 3 with ones more representative. They closely align with what we find in the supplementary figure (Now Supp. Fig. 3).

Several typos in Supp Fig.2 legend on the figure itself (but not in the manuscript).

Line 273: (Figure 2C) should read Fig. 3C.

Discussion:

Lines 303-4: "Treatment with xyloside treatment..."

Line 408: I don't see how you can suggest a different action of LCX on hippocampal

neurons and N2A cells based on MT looping in growth cones, since the latter don't form

a growth cone (at least not in your study).

*Thanks for pointing this out. I have reworded the paragraph.*

Line 442: ...caused changed...

Line 443: predominant effect of actin, or on actin?

Line 452: full stop missing.

Line 454: "...the phenomenology is dose dependent". Normally, phenomenology is a

science (sort of) and cannot be dose dependent.
